# Genetics of asthma: an introduction for the clinician

**DOI:** 10.3402/ecrj.v2.24643

**Published:** 2015-01-16

**Authors:** Simon F. Thomsen

**Affiliations:** Department of Dermatology, Bispebjerg Hospital, Copenhagen NV, Denmark

**Keywords:** association analysis, asthma, epigenetics, genetics, genetic epidemiology, gene discovery, linkage analysis, pharmacogenetics

## Abstract

Asthma runs in families, and children of asthmatic parents are at increased risk of asthma. Prediction of disease risk is pivotal for the clinician when counselling atopic families. However, this is not always an easy task bearing in mind the vast and ever-increasing knowledge about asthma genetics. The advent of new genotyping technologies has made it possible to sequence in great detail the human genome for asthma-associated variants, and accordingly, recent decades have witnessed an explosion in the number of rare and common variants associated with disease risk. This review presents an overview of methods and advances in asthma genetics in an attempt to help the clinician keep track of the most important knowledge in the field.

It has long been known that asthma runs in families and that children of asthmatic parents are at increased risk of asthma. However, asthma is not caused by a single mutation in one gene, and therefore the transmission of the disease through generations does not follow simple Mendelian inheritance typical of classic monogenic diseases, such as Huntington's disease (autosomal dominant) or sickle-cell disease (autosomal recessive). Rather, asthma is a polygenic, multifactorial disorder, which means that many factors contribute to its development. These factors are both genetic and environmental; accordingly, the combined action of several genes interacting with one another and with environmental factors causes the condition (1).

Characteristic of multifactorial disorders is that a person's risk of disease depends on the degree of genetic relatedness between that person and the relative with the disease. Furthermore, the risk is usually higher if the relative is severely affected or if the relative was affected at an early age. Unlike single gene disorders, the asthma phenotype is expressed non-linearly and is highly variable. This makes prediction of asthma status for a given genotype, or combination of genotypes, difficult.

## Genetic epidemiology of asthma

Looking at the population gives clues about asthma being a heritable trait. First, there are large geographic and racial differences in disease occurrence. For example, the prevalence of asthma in many Western populations is high, up to 20%, whereas populations from the developing world exhibit much smaller prevalence rates, some as low as 1% or even lower (2). This is only indicative of a genetic causation in asthma, as different populations also have very different environmental circumstances. Second, offspring of asthmatic parents are at increased risk of asthma (Table 1). The recurrence risk of asthma in children with one affected parent is around 25%, whereas the risk if both parents are affected is around 50%. Twin studies also support asthma being much more likely to occur in an individual if that individual has a genetically close relative with the disease. For instance, the recurrence risk of asthma in monozygotic twins is much higher than in dizygotic twins, highlighting the role of genetic risk factors in asthma (3). Nevertheless, the fact that the concordance for asthma in monozygotic twins is not 100% – but around 75% – points to environmental risk factors also playing an important role.

**Table 1:** Recurrence risk of asthma

Affected relative	Person's own risk of asthma (%)
No family history	5
Uncle/nephew/niece	10
Half sibling	10
Full sibling	25
One parent	25
Dizygotic twin	35
Two parents	50
Monozygotic twin	75

While it is obvious that the individual risk of asthma is dependent on familial background, the phenotypic expression of asthma may be modified by other genetic and environmental factors. It is deemed that a small number of genes set the individual background risk that is acted upon by another set of modifying genes and also environmental factors. For example, individuals with early-onset asthma more frequently have a family history of asthma than do those with later-onset asthma, suggesting that genes influence the age at onset of the disease (4). In addition, the severity of asthma as judged by symptom frequency, level of lung function, degree of airway responsiveness and airway inflammation, aggregates within families, which suggests that if a person has a positive family history of severe asthma, that person is more likely to develop severe asthma (5).

## Gene discovery

There are different experimental approaches to establishing a connection between a gene and a disease such as asthma. The two main options are genetic linkage analysis and genetic (allelic) association analysis (6). Linkage analysis is useful for encircling larger genomic regions within which a susceptibility gene is likely to reside, whereas association analysis can be used to identify specific genotypes, for example, single-nucleotide polymorphisms (SNPs), that are directly associated with the risk of disease.

### Linkage analysis

Linkage analysis is conducted within affected families and exploits the fact that certain genetic loci or alleles in close proximity on the same chromosome have a tendency to be inherited together, that is, to stay together during meiosis. The premise of linkage analysis is the use of genetic *markers* (sequences of nuclear DNA with known location in the genome) positioned along the chromosome. Thus, if a disease is often transmitted to the offspring along with specific markers, it can then be concluded that the gene(s) causing the disease is located close on the chromosome to these markers. In this instance it is important to note that, although multifactorial disorders such as asthma and allergy are regulated by many genetic loci acting in concert, the mode of inheritance can be reduced to Mendelian inheritance at each of these loci. Therefore, the analysis of specific asthma and allergy genes relies on the same principles as the analysis of monogenic traits. Genetic linkage analysis has identified many regions in the genome related to asthma and allergy. Several of the earliest genetic studies of these disorders performed in the 1980s and 1990s used linkage analysis (7). However, linkage analysis identifies relatively large regions of the chromosome dependent on the genomic distance between markers, and such a large fragment of DNA can easily harbour 10 genes of putative importance. For example, the region 5q31–32, which has been linked to atopic diseases in many studies, harbours the *IL4*, *IL13*, *CD14*, and *SPINK5* genes that have been associated with T cell activity and skin morphogenesis (8, 9). Accordingly, to be able to locate the gene itself, denser marker mapping (fine-mapping) or allelic association analysis is warranted.

### Association analysis

Association analysis can be conducted both within families (family-based association) and among non-related individuals, typically in a case–control population. In the classical sense, the genotype frequencies among a population of diseased individuals, for example, asthmatic individuals are compared with the genotype frequencies in a non-diseased control population (healthy controls). If the gene frequencies differ between the cases and the controls, the alleles are said to be associated with the disease. The classical candidate gene approach association analysis is based on knowledge of a specific gene's role in the regulation of a pathogenic process, for instance, one relating to mucosal cell repair or immune mechanisms. An extension to allelic association analysis, which is ‘assumption-free’, is *genome-wide association* (GWA), where numerous genetic variants, often >500,000 SNPs, spanning the entire genome are tested for association with a particular disease. Several GWA studies of asthma and allergy have been conducted and these have identified a number of candidate genes (8, 9).

## Molecular genetics of asthma

Over a hundred different genes have been associated with asthma and the list is still growing. Asthma susceptibility genes fall mainly into three categories relating to 1) functioning of the immune system, 2) mucosal biology and function, and 3) lung function and disease expression (8, 9). However, just because a gene has been associated with asthma in a single study does not necessarily establish a connection between that gene and the disease. A major problem in many genetic studies of asthma is the lack of replication of results from previous studies. Notably, only a subset of identified genes has been found to be associated with asthma in more than one study; and many regard replication as one of the most important features of a gene's candidacy (8, 9). However, some genes may be important only in a subset of asthmatics, for example, in childhood-onset asthma, atopic asthma, house dust mite sensitive asthma, or occupational asthma, and therefore replication across these different populations cannot always be expected. Moreover, some genes are expressed only in certain environmental contexts, for instance, in children growing up with a cat (10) or in those exposed to passive smoking in the first years of life (11, 12). The importance of gene-environment interaction in asthma causation should not be underestimated; every gene's role should be viewed within the context of a permissive environment.

### ADAM33

A disintegrin and metalloproteinase 33 (*ADAM33*) was the first positionally cloned asthma susceptibility gene, meaning that its exact position in the genome was identified before knowing about the function of the gene (13). *ADAM33* is located on chromosome 20p13 and is expressed in bronchial smooth muscle cells and lung fibroblasts. When initially discovered in 2002, it was shown to be strongly linked both to asthma and bronchial hyperresponsiveness. More recently its role has been extended to also involve more subtle aspects of asthma pathogenesis, such as airway remodelling, progression of disease and also chronic obstructive pulmonary disease (14).

### Filaggrin

Filaggrin is a protein that helps maintain an effective skin barrier. Loss-of-function mutations in the filaggrin gene situated on chromosome 1q21 were first associated with the skin disease ichthyosis vulgaris (15) and more recently with atopic dermatitis (16). Mutations are present in a little under 10% of individuals from Western populations and in up to 50% of individuals with atopic dermatitis. It is considered the strongest genetic determinant of atopic dermatitis, increasing the risk about four-fold (17). Moreover, filaggrin mutations have been associated with development of allergic sensitisation, hay fever, and asthma, but only in subjects with atopic dermatitis (18). Interestingly, studies have found that dysfunction of the skin barrier not only enhances sensitisation to allergens but also leads to systemic allergic responses, such as increased IgE levels and airway hyperresponsiveness, indicating that absorption of allergens through the skin of patients with atopic dermatitis is a prerequisite for the development of other allergic conditions, such as asthma and hay fever (19).

### Largest asthma genetics study

The largest and most comprehensive study of asthma genetics to date was conducted in 2010 by a consortium of more than a hundred centres worldwide (20). They ran a GWA study (the GABRIEL study), which genotyped 10,365 persons with asthma and 16,110 unaffected persons to test for association between 582,892 SNPs and asthma. This large study identified genes on chromosomes 2 (*IL1RL1/IL18R1*), 6 (*HLA-DQ*), 9 (*IL33*), 15 (*SMAD3*), 17 (*ORMDL3/GSDMB*), and 22 (*IL2RB*) associated with asthma. The *ORMDL3* gene, in particular, was associatedwith childhood onset, whereas the *HLA-DQ* gene was related to later-onset asthma. Further, the results showed that 38% of all cases of childhood-onset asthma were attributable to a combination of the identified genes.

The study also found an association between serum total IgE and the *HLA-DRB1* gene within the class II region of the major histocompatibility complex (MHC) on chromosome 6. However, this locus was not associated with asthma; most of the identified susceptibility loci for asthma were not associated with IgE, suggesting that elevation of serum total IgE has only a minor role in asthma development.

## Future of asthma genetics

Molecular genetic studies of asthma have contributed considerably to the understanding of the pathogenesis and natural history of the disease. Nevertheless, the entire endeavour has translated only sparsely, if at all, into new treatments or modifiable options for asthma. It is now evident that asthma is regulated by many genes, each contributing only marginally to disease risk (21). Notably, most known asthma susceptibility genes may increase the risk of disease by about a factor of only 1.2 or even less. GWA and deep sequencing studies held the promise of being the ‘design to end all designs’, but these studies also confirmed our ‘worst’ expectations: that asthma genetically, as well as phenotypically, is a very heterogeneous disorder.

### Next-generation sequencing and genomic analysis

The human genome contains about 21,000 genes. The recent complete sequencing of the entire human genome is a landmark endeavour with a profound impact on the ways we diagnose, treat, and possibly also prevent a number of diseases in the future. However, the Human Genome Project relied on only a few individuals, and to grasp the more subtle variation between individuals and to create a ‘reference genome’ for human genetic analysis, the *1000 Genomes Project* was initiated to sequence the genomes of a large number of people and to provide a comprehensive resource of human genetic variation (22). A major challenge for the future use of this resource is the bioinformatic handling and interpretation of such large data output and the implementation in clinical practice and in the genetic counselling of allergic families.

### Personalised medicine and pharmacogenetics

Personalised medicine is becoming increasingly important in the post-genomic era. The idea behind personalised medicine is to predict, based mostly on genetic information, which patients will experience the best response to treatment. As this field advances, it will be possible to individualise pharmacotherapy based on genetic polymorphisms so that certain drugs are administered only to those patients who are most likely to respond, while harmful effects are avoided in patients who are most likely to experience toxicity and adverse reactions (23). Candidate genes for such studies are those encoding receptor proteins and enzymes involved in drug transportation, processing, degradation and excretion. The continuing elucidation of the biological pathways underlying asthma and allergy will help identify new possible targets for intervention. For example, in a randomised study of an experimental drug blocking the IL-4/IL-13 pathway, certain amino acid variations in the IL4 receptor seemed to predict which patients would have the best therapeutic treatment response in terms of increased lung function (24).

### Epigenetics and gene expression analysis

Epigenetics is the study of heritable changes of a disease that are not caused by changes in the nucleotide sequence of the genetic code itself. Epigenetic mechanisms are thought to explain genomic adaption to various environmental influences; epigenetic alterations can contribute to the development of disease, as can genetic alterations. Several molecular mechanisms are involved in epigenetic modification, for example, DNA methylation, post-transcriptional histone modification, and modification of non-coding RNAs. These changes are thought to alter the expressivity of genes, leading in turn to differences in disease expression between individuals. However, little is known about epigenetic mechanisms in asthma and allergy, and studies are currently underway to unravel these (25). The study of monozygotic twins discordant for asthma and allergy can provide valuable clues because epigenetic mechanisms may partly explain the relatively high degree of discordance between monozygotic twins for these diseases (26). Notably, since both monozygotic twins in a twin pair have, in principle, identical genomes and they also tend to share environmental risk factors to a large extent, it is puzzling that in many instances only one twin develops asthma and allergy while the other remains unaffected or affected to a lesser degree. It is possible that environmental exposures unique to the individual twin, for example, commensal microflora, environmental tobacco smoke, dietary factors, and traffic exhaust, induce epigenetic modifications that lead to individual differences in gene expression and therefore also in disease expression. The expression of genes can be studied in living tissue samples using RNA microarrays. Interest is increasing concerning the activity of genes associated with asthma and allergy since the degree of expressivity of a susceptibility gene may influence the clinical severity of disease. Identification of the mechanisms underlying the regulation and expression of these genes could hold the key to understanding the subtle interplay between environment and genetics in the development and progression of asthma and allergy.
